# Supplementary material for: Transformation of social relationships in COVID-19 America: Remote communication may amplify political echo chambers
Source: Sci Adv. 2023 Dec 20;9(51):eadi1540. doi: 10.1126/sciadv.adi1540 (PMC10732520; doi:10.1126/sciadv.adi1540)
Supplement: Supplementary file 1 — Supplementary Text Figs. S1 to S14 Tables S1 to S7 [file sciadv.adi1540_sm.pdf]

Science Advances
AAAS

Supplementary Materials for

Transformation of social relationships in COVID-19 America: Remote communication may amplify political echo chambers

Byungkyu Lee *et al.*

Corresponding author: Byungkyu Lee, bklee@nyu.edu

Sci. Adv. **9**, eadi1540 (2023)
DOI: 10.1126/sciadv.adi1540

This PDF file includes:

Supplementary Text
Figs. S1 to S14
Tables S1 to S7

Supplementary Text

Appendix A. Pretest on network name generators

In online network surveys, the number of boxes presented in the network name generators can affect the number of names provided due to individuals' motivation to "fill in" all the boxes. To avoid such satisfying and heaping effects, existing 2010 and 2016 TESS studies used a one-box design, though this design may take longer as it involves repeating the same questions, "Anyone else?" We conduct a survey experiment as part of the pretest to examine how the number of boxes affects network size, isolation, relationship type, and survey duration, before implementing our main survey.

We randomly assigned respondents to one of six experimental conditions. In the one-box design, we first asked whether they discuss "personally important matters" with others, and then we asked whether they discuss health problems and political matters with others separately. While this design allowed us to differentiate between alters invoked by different name generators, it could be time-consuming. In the multi-box design, we asked whether they discuss "important matters" with others, nudging that important matters may include personal, political, and health-related problems. While this multi-box design could be time efficient, it was difficult to distinguish how they discuss three different matters with the same or different alters. If respondents said they had someone to discuss these matters with, we asked them to provide the first names or initials of their alters. In the one-box design, we asked the follow-up question, "Anyone else?" until we collected five names for the important matters name generators, and three names for the political and health matters name generators, respectively. In the multi-box design, we present two, three, five, seven, or ten boxes, depending on the different experimental conditions, with a one-time follow-up question, "Anyone else"?

Fig. S1, Panel A shows a positive association between the number of boxes presented and network size, but the network size from the one box design is similar to those from the other designs. Panel B shows that the number of boxes does not significantly affect the level of isolation. Panel C shows that the duration of surveys in the one-box design is not significantly different from those from other designs. Panel D confirms that the distribution of relationship types collected by the one-box design is similar to those from other designs, except for the higher proportion of spouse in the seven-box design, the higher proportion of parent in the two-box design, and the higher proportion of coworker in the ten-box design.

Common prompt across all conditions:
Now we are going to ask you some questions about your relationships with other people. We will begin by identifying some of the people you interact with on a regular basis. You may refer to these people in any way you want; for example, you may use just their first names or initials. We are not interested in the identities of these people. We just need to have some way to refer to them so that when we ask you some follow-up questions, we both know whom we are talking about.

In one box design, respondents are first presented with the personally important matters name generator:

Q1. From time to time, most people discuss personally important matters with other people. Looking back over the last month – who are the people with whom you discussed matters personally important to you? Do you have anyone?

Q2. (If Q1 is yes) Please type the first name or initial of one person below. You'll have the chance to tell us about additional people you talk to in the next few questions.

____

If respondents have provided some names, then we ask

Q3. Anyone else?
Yes. Add additional name in the box : _________
No. I have no more names to add.

Repeat Q3 until it collects the five names.

The presentation of health matters (Q4) and political matters (Q5) name generators is randomly ordered.

Q4. From time to time, most people discuss health problems with others. Looking back over the last month, who are the people with whom you discuss your physical, mental, and emotional health matters? Do you have anyone?

Q5. From time to time, most people discuss government, elections, and politics with others. Looking back over the last month, who are the people with whom you discuss political matters? Do you have anyone?

If respondents do not mention one person before,

Q6-1. Please type the first name or initial of one person below. You'll have the chance to tell us about additional people you talk to in the next few questions.

____

If respondents mention at least one person before,

Q6-2. Please type the first name or initial of one person below. He or she may be the same or different from the people on the list you provided earlier. If he or she is the same person, please type the same first name or initial.

____

If respondents have provided some names, then we ask:

Q8. Anyone else?
Yes. Add additional name in the box : _________
No. I have no more names to add.

Repeat Q8 until it collects the three names for each name generator.

In the multi-box design, respondents are first presented with the general important matters name generator.

Q1. From time to time, most people discuss important matters with other people. They may include personal matters, politics, and/or health-related problems. Looking back over the last month – who are the people with whom you discussed matters important to you? Do you have anyone?

Q2. Who are those people with whom you discussed matters important to you? Please type their first names or initials below, one person in a box.

N box	2	3	5	7	10
Response Options					__________

If respondents have filled all boxes, then we ask
Q3. Anyone else?
Yes. Add additional name in the box : _________
No. I have no more names to add.

Panel A. Network size

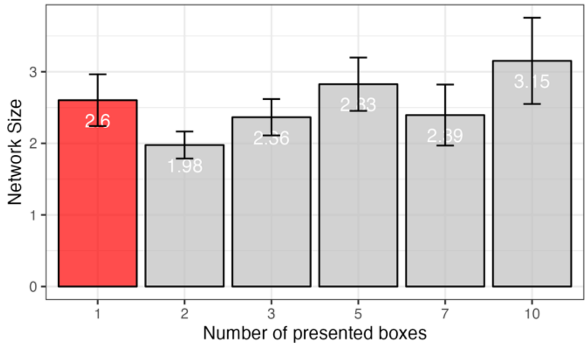

Panel B. Network isolation

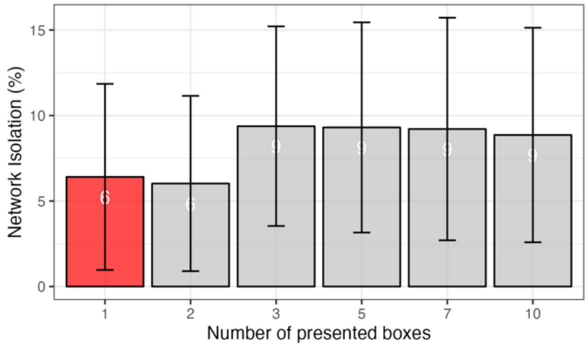

Panel C. Survey length

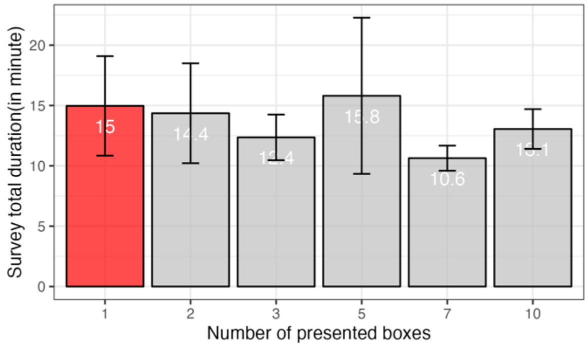

Panel D. Relationship Type

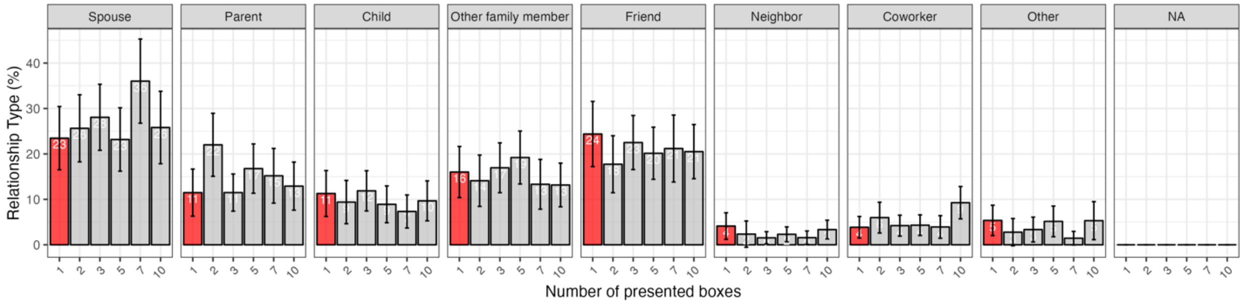

Fig. S1. The comparison of network size, network isolation, survey length, and relationship type by the number of presented boxes.

Appendix B. Introduction to network name generators and name interpreters in the COVID-19 network study and other surveys.

The ego-centric network survey consists of two parts: name generator and name interpreter. The content of network name generators is crucial since it determines which ties people would report as part of their core discussion networks. The earliest nationally representative ego-centric network survey was the General Social Survey in 1985 which uses "important matters" name generators. Since then, numerous surveys have followed a similar format with slightly different wordings (see the bottom of Appendix F for the exact wordings of all network name generators in all eight studies). Based on the pretest results on the number of boxes in network name generators (see Appendix B), we decided to use the multiple generators with a one-box design instead of a single-generator multi-box design. The former, mimicking traditional offline network surveys, enables us to examine tie multiplexity as well.

Here, we first introduce our network name generators, followed by network name interpreters. In our 2020 COVID survey, we employed three name generators that asked with whom people discuss (a) important matters, (b) political matters, and (c) health matters. We asked respondents to type a name (e.g. initials, first names, nicknames) in a box for each network name generator. After collecting up to five names that respondents discuss personally important matters with, we asked them to provide a name in a box again for political matters and health matters name generators, in random order. We specifically ask them to type the same name if they refer to the same person. If they indicated that they had no one to discuss, we then asked why for each network name generator. After collecting all names, we created a unique name list per respondent using Qualtrics' JavaScript and asked about the nature of relationships and characteristics of the alters using the name interpreters. Specifically, the 2020 COVID network survey asked:

Q1. Now we are going to ask you some questions about your relationships with other people. We will begin by identifying some of the people you interact with on a regular basis. You may refer to these people in any way you want; for example, you may use just their first names, nicknames, initials, or relationships with you. We are not interested in the identities of these people. We just need to have some ways to refer to them so that when we ask you some follow-up questions, we both know whom we are talking about.

Respondents are first presented with the personally important matters name generator.

Q2. From time to time, most people discuss personally important matters with other people. Looking back over the last month – who are the people with whom you discussed matters personally important to you? Do you have anyone?

(If Q2 is Yes), Q3. With whom did you discuss important matters? Please type the first name, nickname, or initial of one person below. You'll have the chance to tell us about additional people you talk to in the next few questions.

____

If respondents have provided some names, then we ask,

Q4. Anyone else?
Yes. Add additional name in the box : _________
No. I have no more names to add.

Repeat Q4 until it collects the five names.

The presentation of the following health matters (Q5) and political matters (Q6) name generators is randomly ordered.

Q5 From time to time, most people discuss health-related matters with others. Looking back over the last month, who are the people with whom you discussed your physical, mental, and emotional health? Do you have anyone?

Q6. From time to time, most people discuss government, elections, and politics with others. Looking back over the last month, who are the people with whom you discussed political matters? Do you have anyone?

If respondents do not mention one person before,

Q7-1. With whom did you discuss health-related matters / political matters? Please type the first name, nickname, or initial of one person below. You'll have the chance to tell us about additional people you talk to in the next few questions.
____

If respondents mention at least one person before,

Q7-2. With whom did you discuss health-related matters / political matters? Please type the first name, nickname, or initial of one person below. He or she may be the same or different from the people on the list you provided earlier. If he or she is the same person, please type the exact same name you used before.

____

If respondents have provided some names, then we ask,

Q8. Anyone else?

Yes. Add additional name in the box : _________
No. I have no more names to add.

Repeat Q8 until it collects the three names.

Once we collect all names, then we ask the following set of network name interpreters (Q10-Q17) after expressing our thanks.

Q9. Thanks for providing the names of people with whom you discuss matters that are either personally important, health-related, or political. We will ask you about the nature of your

relationship with them, and their demographic characteristics. Keep in mind that your answers to these questions will be kept confidential and anonymous.

Q10. Thinking back to the most recent discussion you had with them: what did you talk about? Please check all that apply.

Family, Kids and Education	Work and Career	Personal Finance, and Housing	Health	Relationship Issues	Politics	Other topic	Don't remember

Q11. Below is a list of ways that people can be connected to each other. Which of the following best describes your relationship with them? Please choose the only one that best describes each relationship.

Parent	Spouse	Sibling	Child	Other family member	Co-worker	Friend	Neighbor	Other

Q12. Over the last month, have you discussed things that are related to Coronavirus (i.e., COVID-19) with each person?

No	Yes	Don’t remember

Q13. What did you use for your communication the last time you talked to each person? Please check all that apply.

In-person	Telephone	Video Call (e.g., Skype)	Text Message (e.g., WhatsApp)	Email	SNS (e.g., Facebook, twitter)	Other

Q14. What is each person's sex?

Male	Female

Q15. What is each person's race? Please choose the only one that best describes each person's race.

White	Black	Hispanic	Asian	Other	Don't know

Q16. How old is each person?

Younger than 20	20-39	40-59	Older than 60	Don't know

Q17. What is the highest level of education each person has completed?

Less than high school	High school degree or GED	Some college	College degree or higher	Don't know

Q18. Generally speaking, does each person probably think of himself/herself as a Republican, Democrat, Independent, or Something else?

Republican	Democrat	Independent	Something else	Don't know

The 1985, 2004, 2010 General Social Surveys, and the 2010 Time-Sharing Experiment for Social Sciences Study asked:

> "From time to time, most people discuss important matters with other people. Looking back over the last six months—who are the people with whom you discussed matters important to you? Just tell me their first names or initials." IF LESS THAN 5 NAMES MENTIONED, PROBE: "Anyone else?"

The 1992 Cross-National Election Studies asked:

> "From time to time, most people discuss important matters with other people. Looking back over the last six months, I'd like to know the people you talked with about matters that are important to you. Can you think of anyone?" IF LESS THAN 4 NAMES MENTIONED, PROBE: "Is there anyone else you talk with about matters that are important to you?" [AFTER THE SECOND NAME: "Anyone else (you can think of)?"] AFTER THEN, ASKING: "Aside from[n] anyone you have already mentioned, who is the person you talked with most about the events of the recent presidential election campaign?"

The 2008 PEW study asked:

> "From time to time, most people discuss important matters with other people. Looking back over the last six months — who are the people with whom you discussed matters that are important to you? If you could, just tell me their first name or even the initials of their first AND last names." [RECORD UP TO 5 - LOOP. IF LESS THAN 5 MENTIONED, PROBE: "Anyone Else?"] [INTERVIEWER: If R says a relationship instead of a name/initials (e.g. "my wife" or "my brother"), change "my" to "your" – i.e. type as "your wife" or "your brother"] [PROGRAMMER: Also include "No additional mentions" (Code 97), "Don't know" (98) and "Refused" (99) punches]

The 2016 Time-Sharing Experiment for Social Sciences Study asked:

> "From time to time, most people discuss government, elections and politics with other people. Looking back over the last month – who are the people with whom you discussed matters political to you? Just tell us their first names or initials." IF NEITHER OF CHECK BOXES IS CHECKED, DO NOT ALLOW TO PROCEED UNTIL 1 OF THEM IS CHECKED. SHOW THE FOLLOWING PROMPT: "Please check "Add another name" if you would like to add more names or check "I have no more names to add" if you do not want to add any more names."

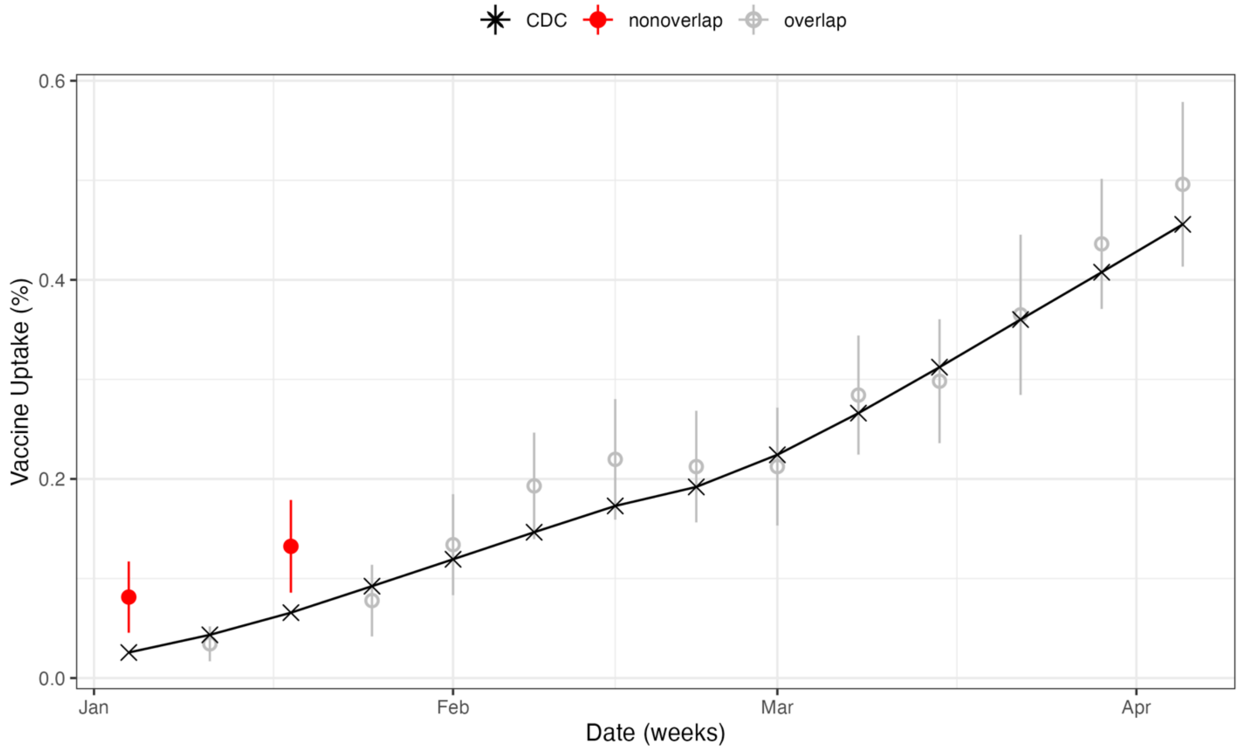

Fig. S2. Estimated COVID-19 vaccination rates from our survey against the CDC's official vaccination rates. Each circle dot represents the estimated weekly vaccination rates with 95% confidence intervals from our survey from 2021 January to 2021 March, adjusted by post-stratified weights derived from survey raking. Each X denotes the weekly vaccination rate from CDC. Red dots indicate when our survey-based estimates failed to predict CDC benchmark vaccination rates with 95% confidence intervals, and grey dots indicate when they overlap.

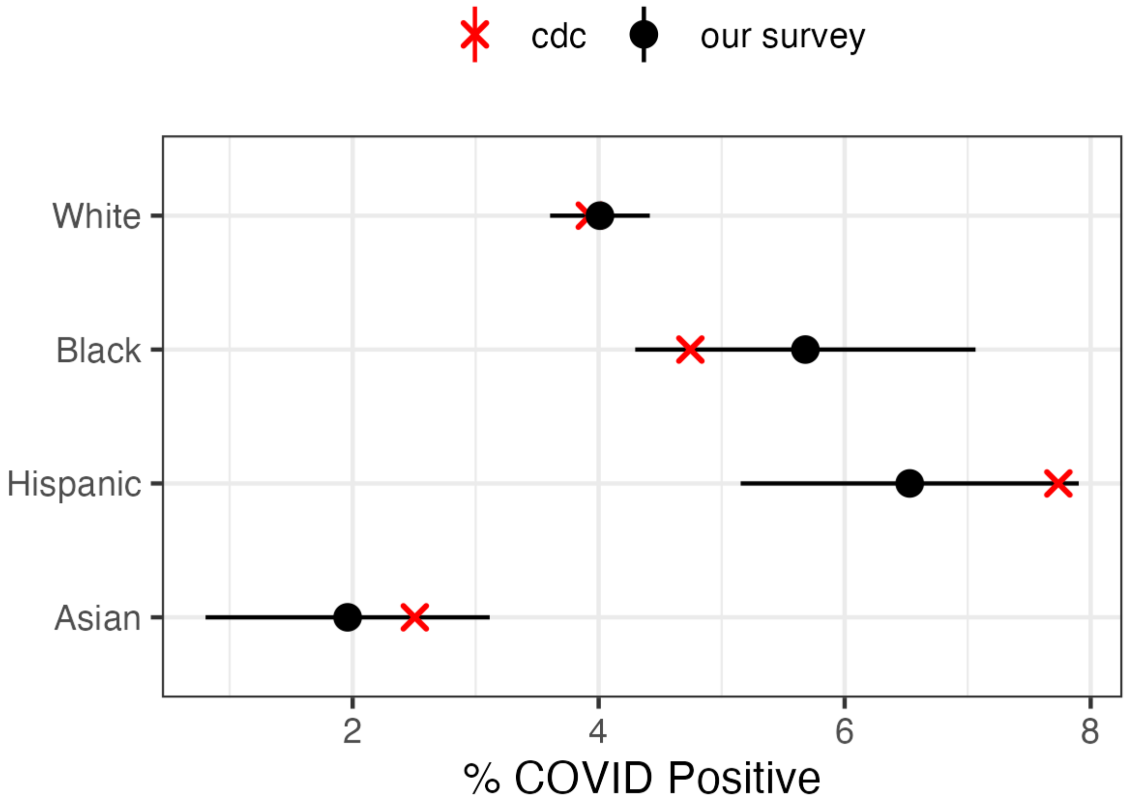

Fig. S3. Estimated race-specific COVID-19 infection rates from our survey against the CDC's official COVID infection rates. Each circle dot represents the estimated COVID-19 vaccination rates with 95% confidence intervals from our survey from 2020 April to 2021 March, adjusted by post-stratified weights derived from survey raking. Each X denotes the COVID-19 positivity rates from CDC during the same period.

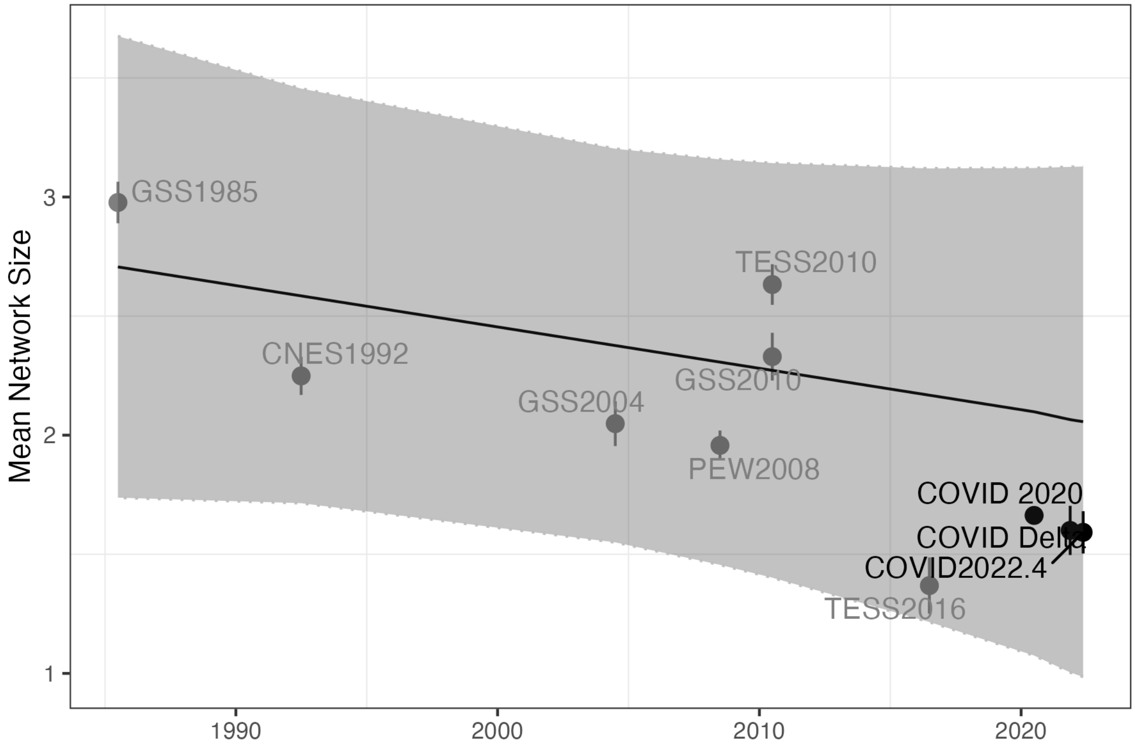

Fig. S4. The trends in the size of core discussion networks from 1985 to 2022 while excluding the 2004 GSS and 2016 TESS studies in the benchmark. Network sizes are capped at five for effective comparison across different surveys (i.e., the maximum network size in the 1992 CNES data was five). Weighted means for network size with 95% confidence intervals are presented. The 95% confidence intervals for average network size in 2020 are very narrow due to the large sample size. The grey box shows the benchmark network size and 95% confidence intervals from the meta-analysis that exclude the 2004 GSS and 2016 TESS data to assess the impact of outliers.

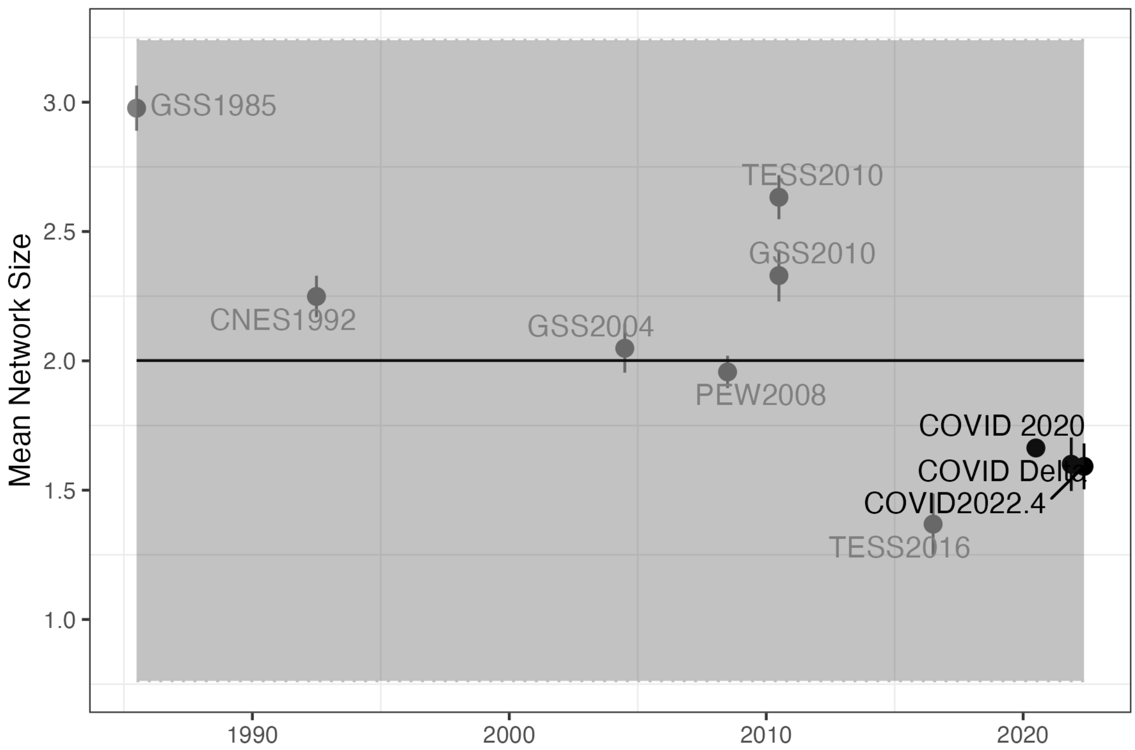

Fig. S5. The trends in the size of core discussion networks from 1985 to 2022 while only using the 2010 and 2016 TESS studies in the benchmark. Network sizes are capped at five for effective comparison across different surveys (i.e., the maximum network size in the 1992 CNES data was five). Weighted means for network size with 95% confidence intervals are presented. The 95% confidence intervals for average network size in 2020 are very narrow due to the large sample size. The grey box shows the benchmark network size and 95% confidence intervals from the meta-analysis including only the 2010 and 2016 TESS surveys that use the same web-based design with the COVID network survey. As it is not feasible to include a linear trend term in the meta-analysis with only two observations, we calculate the mean and 95% confidence intervals for the benchmark.

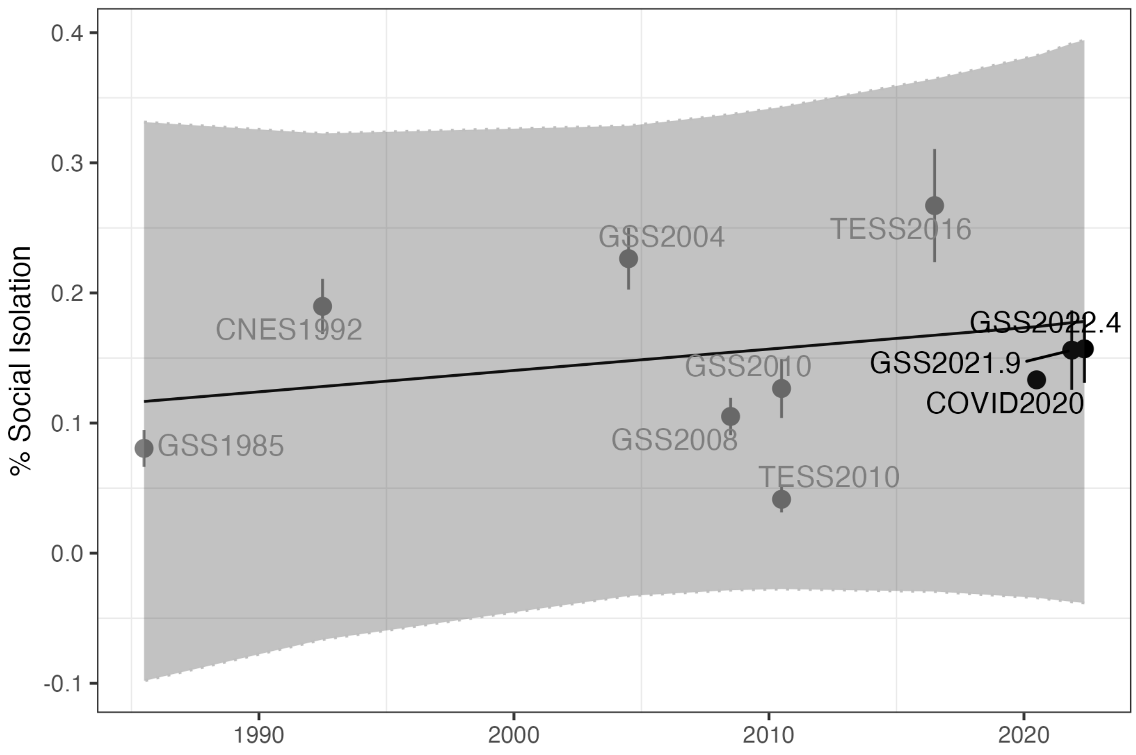

Fig. S6. Trends in social isolation from 1985 to 2022. Weighted means for social isolation with 95% confidence intervals are presented. The 95% confidence intervals for social isolation in 2020 are very narrow due to the large sample size. The grey box shows the mean social isolation and 95% confidence intervals from a meta-analysis of the social isolation estimates from 1985 to 2016.

Panel A. The distribution of race category

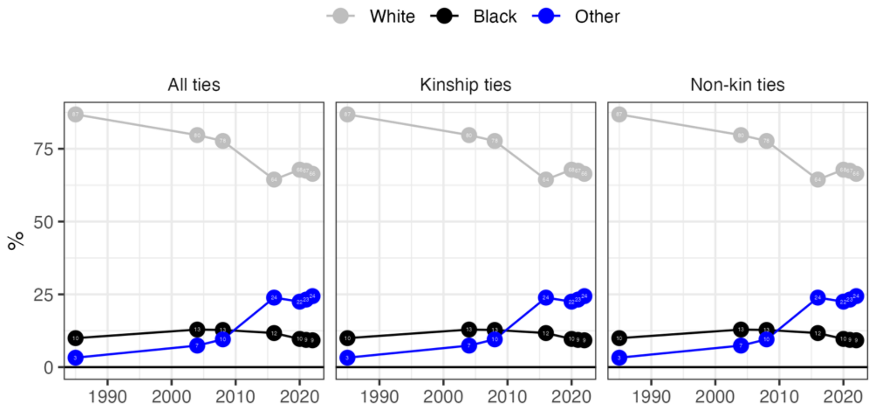

Panel B. The level of absolute homophily: the % same race

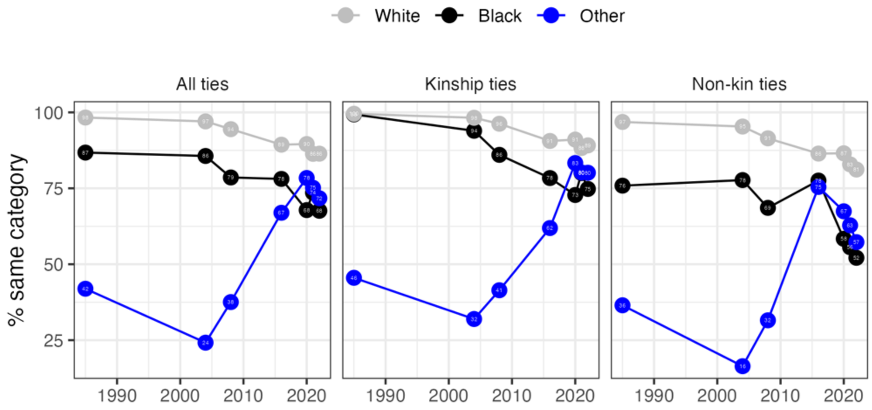

Panel C. The level of choice homophily: Coleman index: from -1 (heterophily) to 1 (homophily)

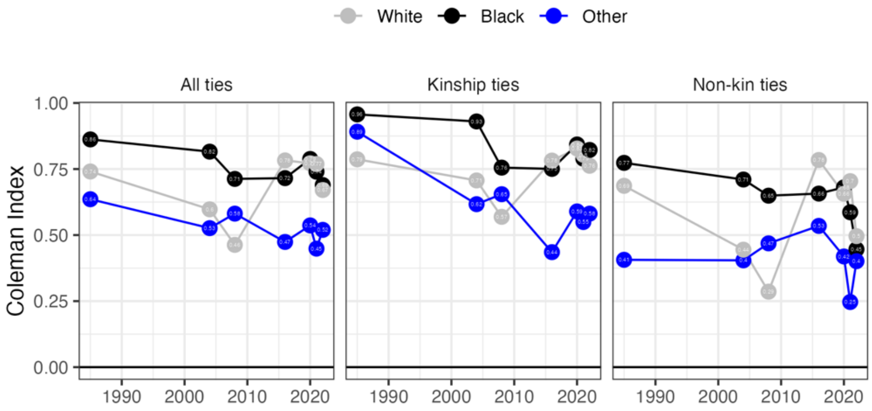

Fig. S7. The decomposition of racial homophily across different racial groups from 1985 to 2022. Panel A shows the distribution of each race category over time, which has been used to control for structural opportunities in calculating the Coleman index (Panel C). Panel B shows the level of absolute homophily measured by the proportion of the same race ties. Panel C shows the level of choice homophily measured by the Coleman index.

Panel A. The distribution of partisanship category

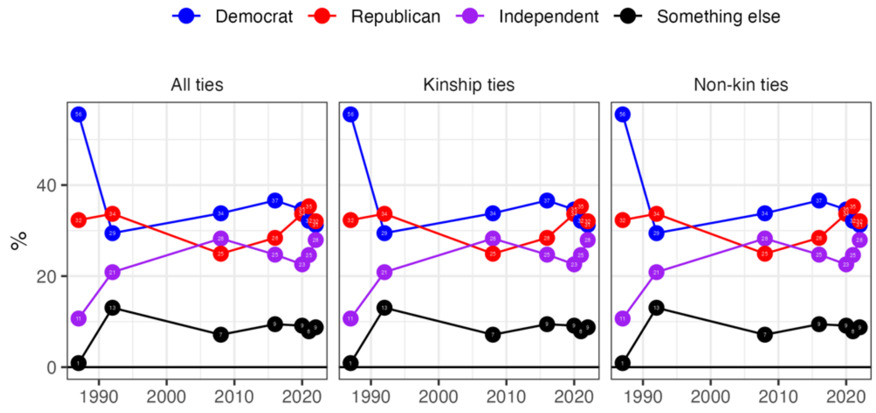

Panel B. The level of absolute homophily: the % same partisanship

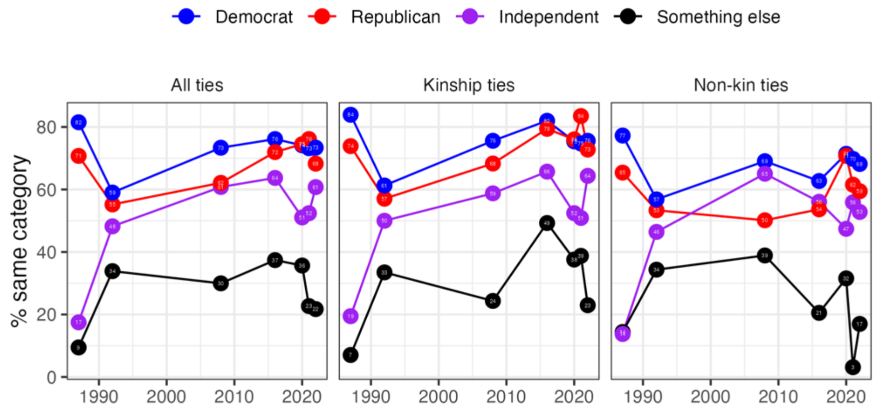

Panel C. The level of choice homophily: Coleman index: from -1 (heterophily) to 1 (homophily)

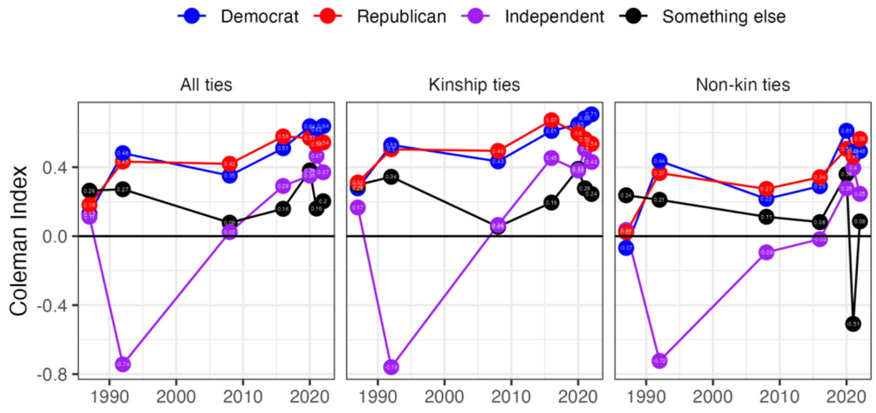

Fig. S8. The decomposition of political homophily across different partisan groups from 1987 to 2022. Panel A shows the distribution of each partisanship category over time, which has been used to control for structural opportunities in calculating the Coleman index (Panel C). Panel B shows the level of absolute homophily measured by the proportion of the same partisan ties. Panel C shows the level of choice homophily measured by the Coleman index.

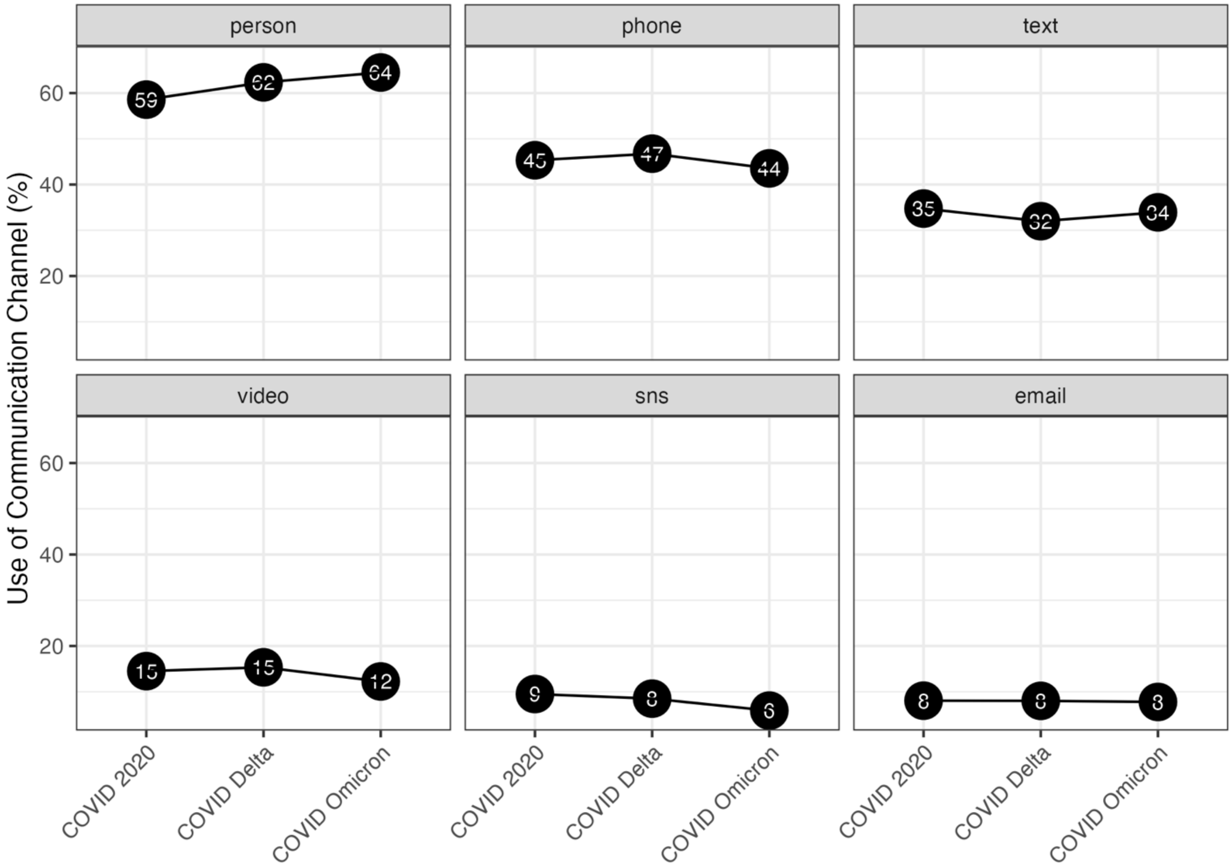

Fig. S9. The patterns of communication channels during the COVID-19 pandemic. Weighted proportions with 95% confidence intervals for channel usage with confidants are presented across three phases of the COVID-19 pandemic during the study period.

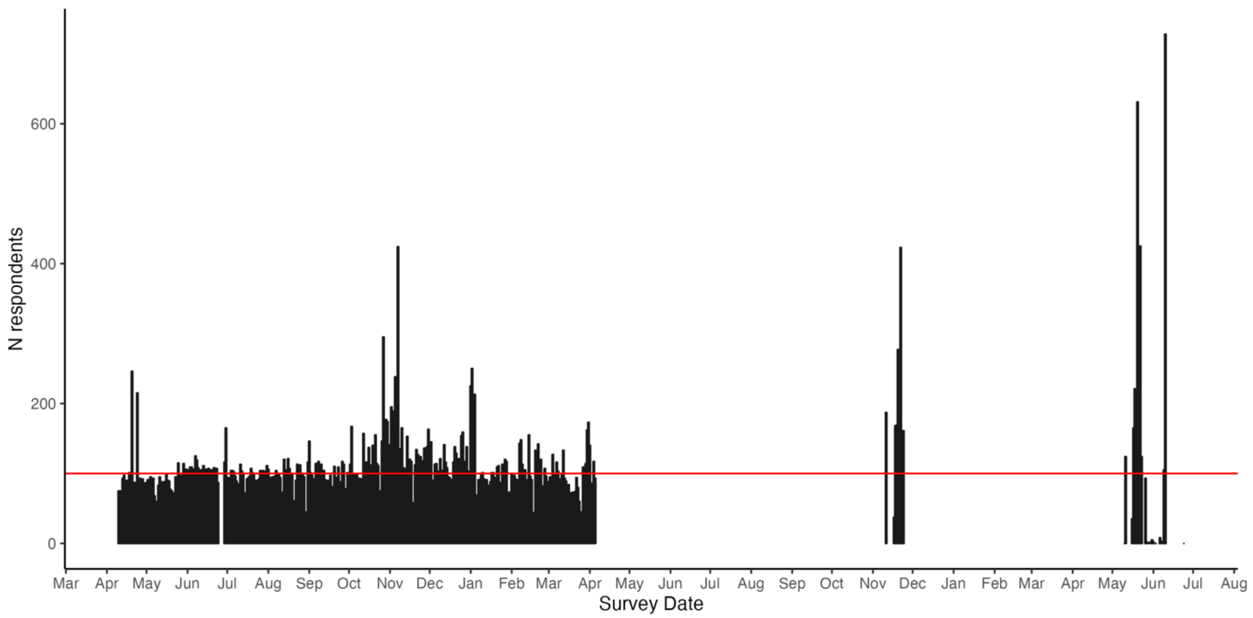

Fig. S10. The over-time distribution of sample sizes during the entire survey period. The total number of respondents in the first survey's analytic sample is 36,345 for 357 days from April 10, 2020, to April 5, 2021, which yielded 101.8 competes per day. Here, survey responses for four days (June 25, 26, 27, and 28 in 2020) are missing due to updates on new survey items. We had more completed responses several days in November 2020 and January 2021, though we carefully examined that their demographic characteristics are not significantly different from other cases in the same month. We did not have any daily complete target in the second and third surveys, where survey respondents had been recruited for two weeks in November 2021 (N = 1,776) and for a month in May 2022 (2,912).

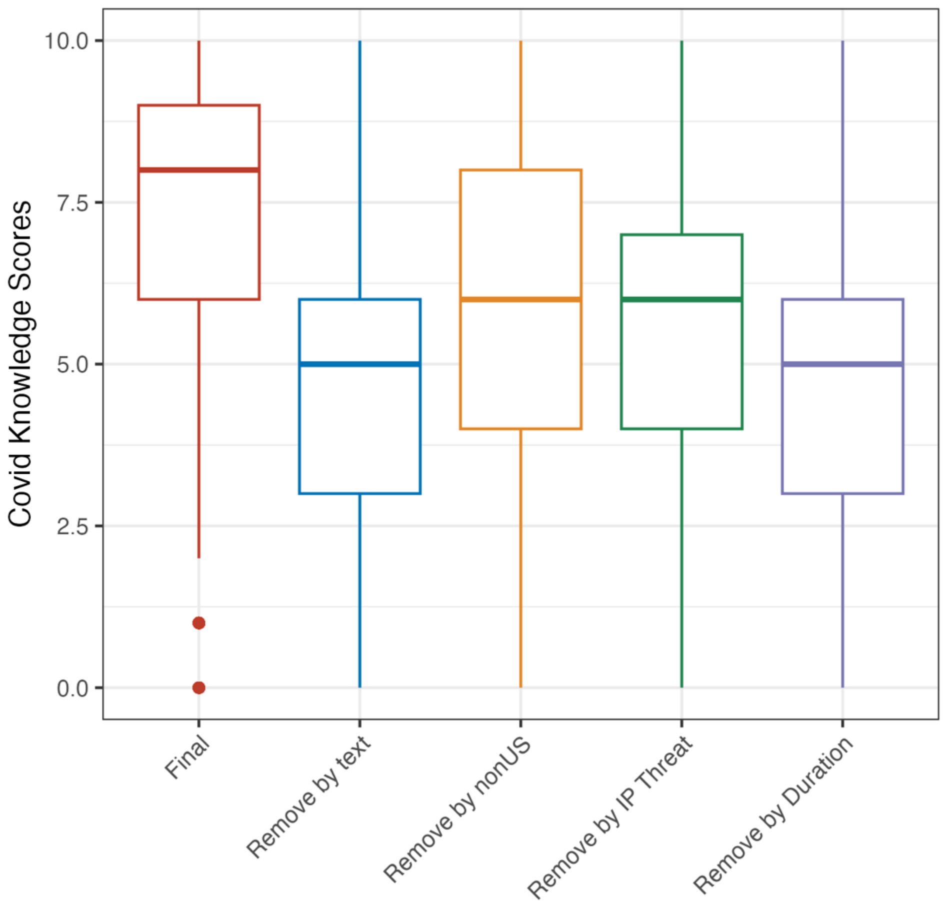

Fig. S11. COVID-19 knowledge scores across different quality control criteria. To validate our quality control procedure, we compared the level of knowledge on COVID-19 that we created based on World Health Organization's guideline (https://www.who.int/emergencies/diseases/novel-coronavirus-2019/advice-for-public/myth-busters. Accessed as of April 20, 2020) , "Coronavirus disease (COVID-19) advice for the public: Mythbusters." We asked the following ten questions about COVID-19 and counted the number of True answers for each person: 1. 5G mobile networks spread COVID-19. (False) 2. Antibiotics are effective in preventing and treating the new coronavirus. (False) 3. Drinking alcohol will protect you against COVID-19. (False) 4. Everybody who gets COVID-19 shows symptoms. (False) 5. Eating garlic helps prevent infection with the new coronavirus. (False) 6. The new coronavirus affects only older people. (False) 7. You cannot recover from the coronavirus disease (COVID-19). (False) 8. The new coronavirus can be transmitted through mosquito bites. (False) 9. COVID-19 virus can be transmitted in areas with hot and humid climates. (True) 10. Vaccines against pneumonia cannot protect you against the new coronavirus. (True).

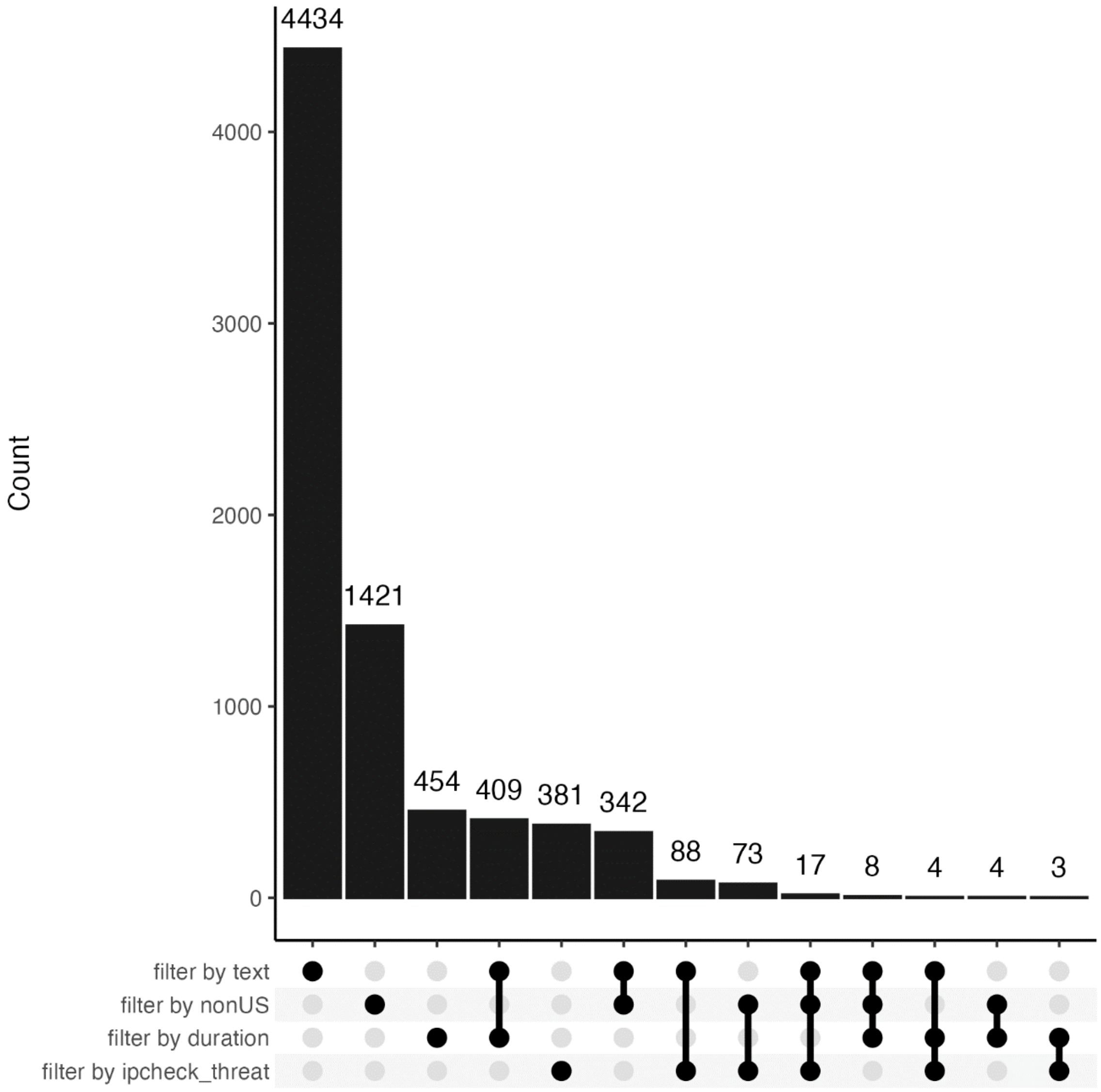

Fig. S12. The joint distribution of fraudulent responses by different quality control types.

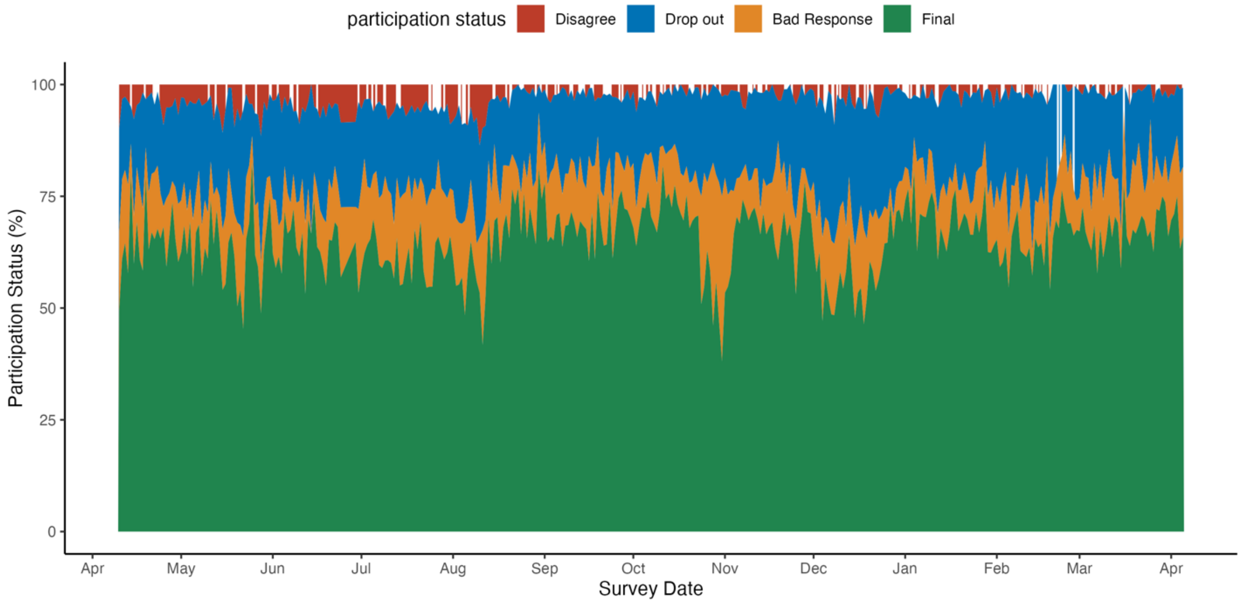

Fig. S13. The trends of participation status from April 2020 to April 2021. Among 56,280 individuals who opened the survey link in the entire period in our first survey, 3.2% did not agree to participate, 19.6% dropped out in the middle of the survey, 12.5% were classified as bad/fraudulent responses, and 64.6% consisted of the analytic sample in our first survey. In the second survey, among 2,506 individuals who opened the survey link, 2.7% did not agree to participate, 14.0% dropped out in the middle of the survey, 12.4% were classified as bad/fraudulent responses, and 70.9% consisted of the analytic sample. In the third survey, among 4,222 individuals who opened the survey link, 3.4% did not agree to participate, 20.9% dropped out in the middle of the survey, 6.8% were classified as bad/fraudulent responses, and finally 69.0% consisted of the analytic sample.

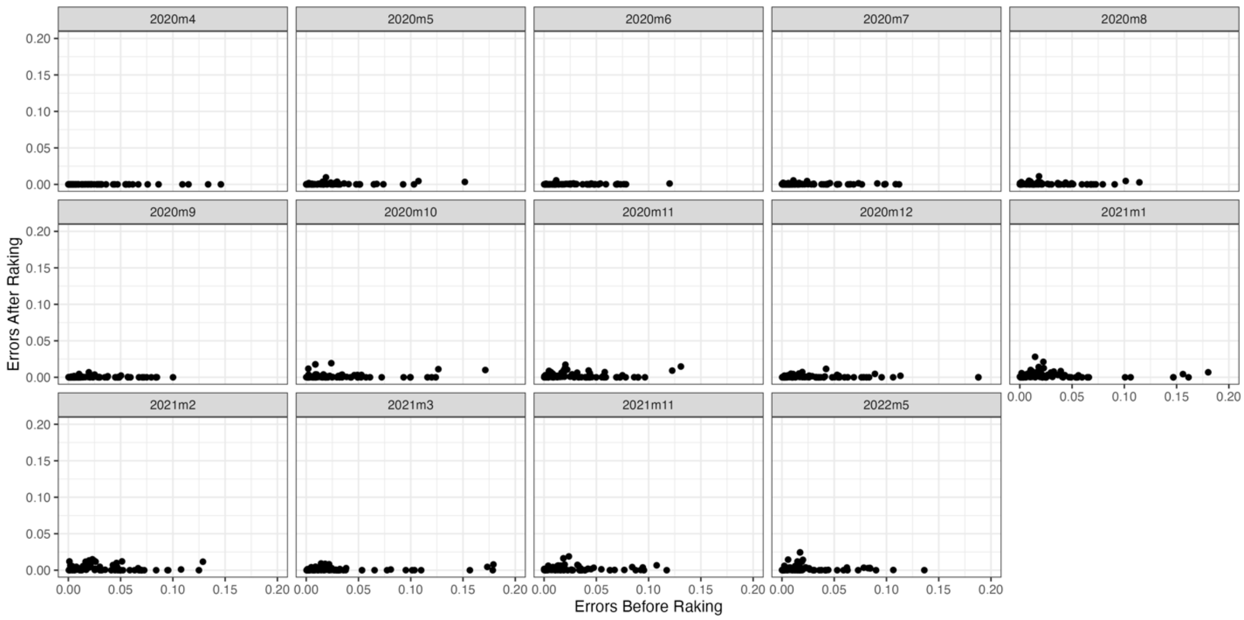

Fig. S14. Changes in sampling bias for demographic variables before and after raking. We calculate sampling bias for 10 variables and 98 categories used in the raking procedure by taking the absolute difference in the proportion of each variable from our sample against that from current population survey (CPS) data. Each dot represents the amount of bias for a variable before (x-axis) and after (y-axis). The mean biases across all months before and after raking are 0.023 and 0.001 respectively.

Table S1. The distribution of network sizes across different surveys.

Data set	GSS	GSS	CNES	GSS	PEW	GSS	TESS	TESS	COVID	COVID 2021	COVID 2022
Year	1985	1987	1992	2004	2008	2010	2010	2016	2020/21	Nov	May
% of Isolation	8.1	19	22.6	10.5	12.7	4.1	26.7	13.3	15.6	15.7	8.1
Size = 1	14.8	13.7	19.8	35.5	21.4	31.2	37.6	41.5	40.2	41.5	14.8
Size = 2	14.7	20.7	19.8	23.5	23.1	17	17.8	24	26.9	23.9	14.7
Size = 3	21.6	16.6	17.1	15.7	19.6	14.9	11.5	12	7.5	9.4	21.6
Size = 4	15.4	30	9.2	8	9.4	10.4	2.9	5.1	5.5	5.8	15.4
Size = 5	20		6.6	6.9	7.7	7.1	2.4	1.9	1.3	1.4	20
Size = 6	5.4		4.8		6.1	5.2	1.2	2.1	3	2.3	5.4
Size = 6 +						10.1					
Network Size											
Max	6	4	6	5	6	70	6	6	6	6	6
Mean	3.03146601	2.24886191	2.09697266	1.95713191	2.39037156	3.27305228	1.3801565	1.68442585	1.63028437	1.61505361	3.03146601
Mean (capped at six)	3.03	2.25	2.1	1.96	2.39	2.84	1.38	1.68	1.63	1.62	3.03
Mean (capped at five)	2.97700029	2.24886191	2.04853845	1.95713191	2.32971588	2.6325218	1.36860747	1.66314763	1.6001526	1.59211264	2.97700029
N	1531	1318	1426	2128	1272	2061	526	36345	1776	2912	1531
Survey Period	spring	spring	fall	fall	fall	summer	spring	fall	all seasons	fall	spring
Presidential Election	no	no	yes	yes	no	no	no	yes	yes	no	no
Survey Mode	FF/tel.	FF/tel.	tel.	FF/tel.	tel.	FF/tel.	internet	internet	internet	internet	internet

Note. FF: face-to-face, tel: telephone.

Table S2. Relationship compositions in core discussion networks across different surveys from 1985 to 2022.

Dataset	GSS	CNES	GSS	PEW	GSS	TESS	TESS	COVID	COVID	COVID
Year	1985	1992	2004	2008	2010	2010	2016	2020/21	2021 Nov	2022 May
Parent	12.3		13.2	10.9	13.4	12.2	12.5	12.8	12.2	10.9
Sibling	9		9.1	10.8	10.3	9.9	3.8	9.9	9.9	10.4
Spouse	14.5	15.1	19.2	17.4	17.3	22.3	49	25.6	26.7	26.3
Child	9		8.1	12.4	10.2	6.7	6.8	11.7	9.8	10.7
Other family	8.4	34.7	7.2	10.7	6.6	7	3.7	7.4	6.9	6.6
Coworker	8	8.1	7.6	6.8	6	3.7	5.4	3.8	3.4	3.2
Friend	27.7	33	26.6	24	25.8	32.1	16.3	23.2	23.3	24.8
Neighbor	3.1	4.7	2	1.2	1.6	0.9	0.7	1.6	1.9	1.7
Other	8	4.4	7.1	5.7	8.8	5.2	1.8	4	5.9	5.6

Note. To account for the fact that multiple responses are allowed for the GSS and CNES studies, we run 1000 random selections of relationship categories and take the average across 1000 runs in 1985, 1992, 2004, 2008, and 2010. Specifically, we use kin-based random selection: first randomly select one relationship category among kin, and then select one relationship category among other categories, based on the assumption that people would prioritize kin ties over non-kin ties. The mean proportion of each category across 100 simulations is presented here.

Table S3. Comparison of the distribution of network ties by alters' location and the use of in-person channels between the 2008 PEW survey and our COVID survey.

Channel	Location	COVID 2020	COVID Delta (2021 Nov)	COVID Omicron (2022 May)	PEW-a	PEW-b	PEW-c
In-Person	Same household	32.7	32.3	31.4	21.8	21.8	21.8
Not in-person	Same household	3.2	4.4	2.7	0.1	0	0
In-Person	Different household	23.9	27.8	29.1	40.4	54.7	64.5
Not in-person	Different household	40.1	35.5	36.8	37.7	23.4	13.6

Note. The total number of dyads is 4,259 for the 2008 PEW survey and 71,935 for our COVID survey (64,220 for COVID 2020, 2,958 for COVID Delta wave, and 4,757 for COVID Omicron wave). Both the 2008 PEW survey and our COVID survey collect information on whether their alters lived in the same household similarly. In contrast to our COVID survey that asks whether respondents used an in-person channel the last time they talked to their alters, the 2008 PEW survey asks how often respondents had a face-to-face conversation with their alters over the last six months with the response options: several times a day (=1), once a day (=2), several times a week (=3), once a week (=4), once a month (=5), less often (=6), and never (=7). Here, we show results from comparisons with different thresholds to estimate the use of in-person channels in the last conversation in the PEW 2008 survey: a = 1 to 3, b = 1 to 4, c = 1 to 5. We discuss version b (PEW-b) in the manuscript.

Table S4. Results from logistic regression models predicting political homophily across kin and non-kin ties across three phases of the pandemic.

Period	COVID 2020	COVID 2020	All	All
Sample	Kin	Nonkin	Kin	Nonkin
Model	Model1	Model2	Model3	Model4
inperson only	0.0571	-0.185*	0	0
	(0.0572)	(0.0874)	(.)	(.)
remote and inperson	0	0	-0.0688	0.179*
	(.)	(.)	(0.0569)	(0.0869)
remote only	-0.0809	0.0129	-0.147*	0.199*
	(0.0639)	(0.0721)	(0.0636)	(0.0784)
COVID 2020 (p1)			0	0
			(.)	(.)
COVID 2021 (p2)			0.0620	0.0727
			(0.166)	(0.279)
COVID 2022 (p3)			0.0418	-0.363
			(0.168)	(0.284)
p1 X inperson only			0	0
			(.)	(.)
p1 X remote and inperson			0	0
			(.)	(.)
p1 X remote only			0	0
			(.)	(.)
p2 X inperson only			0	0
			(.)	(.)
p2 X remote and inperson			0.194	0.109
			(0.279)	(0.384)
p2 X remote only			-0.0941	-0.276
			(0.234)	(0.357)
p3 X inperson only			0	0
			(.)	(.)
p3 X remote and inperson			-0.186	0.444
			(0.230)	(0.364)
p3 X remote only			0.000712	-0.147
			(0.217)	(0.332)
Male	0.00857	0.141*	-0.0286	0.120+
	(0.0513)	(0.0664)	(0.0489)	(0.0636)
Female	0	0	0	0
	(.)	(.)	(.)	(.)
Race = White	0	0	0	0
	(.)	(.)	(.)	(.)
Black	0.372***	0.551***	0.386***	0.525***
	(0.0918)	(0.118)	(0.0878)	(0.114)

Asian	-0.169	-0.291+	-0.164	-0.326*
	(0.113)	(0.160)	(0.107)	(0.150)
Other	-0.123	-0.318+	-0.158	-0.339*
	(0.161)	(0.171)	(0.149)	(0.163)
Hispanic	0.00854	-0.197+	-0.0229	-0.195+
	(0.0786)	(0.105)	(0.0751)	(0.0998)
Age -29	-0.304***	0.0563	-0.347***	0.0427
	(0.0851)	(0.122)	(0.0799)	(0.115)
Age 30-39	0	0	0	0
	(.)	(.)	(.)	(.)
Age 40-49	0.238**	0.0939	0.160*	0.0521
	(0.0819)	(0.0974)	(0.0785)	(0.0934)
Age 50-59	0.301***	-0.0892	0.213**	-0.109
	(0.0867)	(0.106)	(0.0817)	(0.102)
Age 60-69	0.290**	0.158	0.258**	0.0958
	(0.0905)	(0.119)	(0.0874)	(0.114)
Age 70+	0.565***	0.216	0.487***	0.132
	(0.114)	(0.144)	(0.108)	(0.137)
Less than high school	-0.0879	0.0386	-0.0945	0.0632
	(0.118)	(0.170)	(0.113)	(0.162)
HS	0	0	0	0
	(.)	(.)	(.)	(.)
Some college	-0.0367	0.123	-0.0412	0.0708
	(0.0631)	(0.0903)	(0.0601)	(0.0863)
BS+	0.123+	0.327***	0.0885	0.296***
	(0.0710)	(0.0912)	(0.0674)	(0.0868)
Household Income less than 10K	0.0665	-0.0858	-0.00143	-0.0366
	(0.121)	(0.148)	(0.114)	(0.143)
10K-	-0.00206	-0.275*	-0.0215	-0.278*
	(0.105)	(0.137)	(0.0996)	(0.130)
20K-	0.0680	-0.0252	-0.00182	-0.0730
	(0.0875)	(0.125)	(0.0831)	(0.118)
30K-	0.0351	-0.177	0.0224	-0.0619
	(0.0904)	(0.114)	(0.0862)	(0.108)
40K-	0.0819	-0.146	0.0612	-0.0534
	(0.0885)	(0.121)	(0.0851)	(0.118)
50K-	0.107	0.00409	0.130	0.00129
	(0.0879)	(0.117)	(0.0828)	(0.113)
60K-	0	0	0	0
	(.)	(.)	(.)	(.)
100K-	-0.0368	0.106	-0.0488	0.0575
	(0.0798)	(0.101)	(0.0772)	(0.0972)
150K-	0.252*	0.0977	0.272**	0.106
	(0.0989)	(0.113)	(0.0937)	(0.108)

Married	0	0	0	0
	(.)	(.)	(.)	(.)
Widowed	-0.186	-0.205	-0.211+	-0.261+
	(0.115)	(0.147)	(0.110)	(0.141)
Divorced	-0.282***	-0.220*	-0.251**	-0.245*
	(0.0844)	(0.103)	(0.0804)	(0.0970)
Separated	-0.566***	-0.373*	-0.484**	-0.312+
	(0.158)	(0.187)	(0.151)	(0.177)
Never married	-0.0671	-0.158	-0.0463	-0.152
	(0.0721)	(0.105)	(0.0682)	(0.0997)
DK	-0.0191	-0.159	-0.0202	-0.198
	(0.238)	(0.308)	(0.229)	(0.295)
Household size = 1	0	0	0	0
	(.)	(.)	(.)	(.)
2	-0.0985	-0.0103	-0.128+	-0.0703
	(0.0787)	(0.101)	(0.0752)	(0.0959)
3	-0.0537	-0.106	-0.0545	-0.0799
	(0.0904)	(0.113)	(0.0860)	(0.107)
4	-0.0230	-0.0854	-0.0481	-0.0625
	(0.0972)	(0.120)	(0.0924)	(0.116)
5	-0.114	0.0112	-0.177	0.0269
	(0.115)	(0.154)	(0.109)	(0.149)
more than 5	-0.149	-0.202	-0.137	-0.208
	(0.127)	(0.161)	(0.120)	(0.153)
Republican	0	0	0	0
	(.)	(.)	(.)	(.)
Democrat	0.165**	0.238***	0.207***	0.201**
	(0.0575)	(0.0690)	(0.0551)	(0.0665)
Independent	-1.058***	-1.003***	-1.003***	-0.992***
	(0.0625)	(0.0878)	(0.0597)	(0.0838)
Something else	-1.940***	-1.743***	-1.952***	-1.861***
	(0.128)	(0.224)	(0.121)	(0.216)
Not working now	-0.339	0.132	-0.274	-0.0124
	(0.207)	(0.327)	(0.195)	(0.304)
Other	-0.173*	0.106	-0.156*	0.0861
	(0.0757)	(0.107)	(0.0719)	(0.102)
Retired	-0.150+	0.0524	-0.153+	0.0949
	(0.0833)	(0.110)	(0.0792)	(0.104)
Unable to work	-0.452**	-0.249	-0.425**	-0.269
	(0.142)	(0.290)	(0.140)	(0.273)
Unemployed	-0.312***	-0.0247	-0.311***	-0.0708
	(0.0901)	(0.115)	(0.0876)	(0.112)
Working now	0	0	0	0
	(.)	(.)	(.)	(.)

metro	-0.0152	0.0973	-0.0219	0.106
	(0.0635)	(0.0906)	(0.0610)	(0.0874)
Same household	0	0	0	0
	(.)	(.)	(.)	(.)
Same neighborhood	-0.320***	-0.325**	-0.352***	-0.368***
	(0.0699)	(0.111)	(0.0664)	(0.108)
Same state	-0.444***	-0.445***	-0.443***	-0.467***
	(0.0635)	(0.111)	(0.0611)	(0.107)
Somewhere else	-0.571***	-0.691***	-0.564***	-0.717***
	(0.0757)	(0.124)	(0.0716)	(0.119)
Constant	0.856***	0.400+	1.010***	0.332
	(0.146)	(0.230)	(0.140)	(0.220)
Observations	40912	22143	45760	24787

Note. Standard errors are in parenthesis (+ p < 0.1, * p < 0.05, ** p < 0.01, *** p < 0.005).

Table S5. Demographic comparison between the fraudulent sample and the final sample.

	filtered	final	p
n	7638	41033	
sex = Female (%)	2710 (35.5)	24800 (60.4)	<0.001
age (mean (SD))	33.94 (11.11)	45.63 (16.90)	<0.001
race (%)			<0.001
White	4895 (64.1)	31196 (76.0)	
Black	1325 (17.3)	3976 (9.7)	
Asian	468 (6.1)	1547 (3.8)	
Other	261 (3.4)	1375 (3.4)	
Hispanic	689 (9.0)	2939 (7.2)	
partyid4 (%)			<0.001
Republican	3300 (43.2)	14376 (35.0)	
Democrat	2578 (33.8)	15935 (38.8)	
Independent	1238 (16.2)	8106 (19.8)	
Something else	522 (6.8)	2616 (6.4)	
educ4 (%)			<0.001
Less than high school	394 (5.2)	1516 (3.7)	
HS	1472 (19.3)	9497 (23.1)	
Some college	1565 (20.5)	13552 (33.0)	
BS+	4207 (55.1)	16468 (40.1)	
marital_status (%)			<0.001
Married	4389 (59.4)	20464 (50.7)	
Widowed	270 (3.7)	2120 (5.2)	
Divorced	373 (5.1)	4965 (12.3)	
Separated	280 (3.8)	961 (2.4)	
Never married	2072 (28.1)	11886 (29.4)	
empstat (%)			<0.001
Not working now	242 (3.2)	702 (1.7)	
Other	785 (10.3)	4795 (11.7)	
Retired	285 (3.7)	7409 (18.1)	
Unable to work	151 (2.0)	2646 (6.4)	
Unemployed	802 (10.5)	4666 (11.4)	
Working now	5373 (70.3)	20815 (50.7)	
census_division (%)			<0.001
East North Central	826 (10.9)	5761 (14.0)	
East South Central	316 (4.2)	2547 (6.2)	
Mid-Atlantic	1730 (22.9)	6577 (16.0)	
Mountain	445 (5.9)	3295 (8.0)	
New England	258 (3.4)	2189 (5.3)	
Pacific	1244 (16.5)	5091 (12.4)	
South Atlantic	1731 (22.9)	8760 (21.4)	
West North Central	318 (4.2)	2581 (6.3)	

West South Central	691 (9.1)	4227 (10.3)	
metro = 1 (%)	6153 (80.6)	33979 (82.8)	<0.001
income (%)			<0.001
Less than $10,000	905 (11.8)	3685 (9.0)	
$10,000 to $19,999	629 (8.2)	3709 (9.0)	
$20,000 to $29,999	732 (9.6)	4651 (11.3)	
$30,000 to $39,999	520 (6.8)	4158 (10.1)	
$40,000 to $49,999	421 (5.5)	3410 (8.3)	
$50,000 to $59,999	512 (6.7)	3488 (8.5)	
$60,000 to $69,999	310 (4.1)	2347 (5.7)	
$70,000 to $79,999	487 (6.4)	2630 (6.4)	
$80,000 to $89,999	215 (2.8)	1495 (3.6)	
$90,000 to $99,999	432 (5.7)	1914 (4.7)	
$100,000 to 149,999	1285 (16.8)	4981 (12.1)	
$150,000 or more	1078 (14.1)	3758 (9.2)	
Prefer not to tell	112 (1.5)	807 (2.0)	
household_size (%)			<0.001
1	1652 (21.6)	8360 (20.4)	
2	1488 (19.5)	13280 (32.4)	
3	1708 (22.4)	7591 (18.5)	
4	1850 (24.2)	7284 (17.8)	
5	577 (7.6)	2799 (6.8)	
more than 5	363 (4.8)	1719 (4.2)	

Note. P-value is based on the Chi-square tests.

Table S6. Summary of sample characteristics before and after raking and the target statistics from CPS data in March 2021.

Sample	unweighted	weighted	CPS (target)	Diff unweighted	Diff weighted
N	3302	3302	254736187.3		
Female	1893.0 (57.3)	1716.8 (52.0)	131613078.6 (51.7)	-5.6	-0.3
Age Group					
Age -29	303.0 (9.2)	617.3 (18.7)	54870055.8 (21.5)	12.3	2.8
Age 30-39	884.0 (26.8)	541.8 (16.4)	43692842.2 (17.2)	-9.6	0.8
Age 40-49	541.0 (16.4)	504.8 (15.3)	39228128.3 (15.4)	-1	0.1
Age 50-59	412.0 (12.5)	536.2 (16.2)	41007634.0 (16.1)	3.6	-0.1
Age 60-69	653.0 (19.8)	546.2 (16.6)	38754387.6 (15.2)	-4.6	-1.4
Age 70+	507.0 (15.4)	554.1 (16.8)	37183139.3 (14.6)	-0.8	-2.2
Race					
White	2785.0 (84.3)	2195.7 (66.5)	158466542.8 (62.2)	-22.1	-4.3
Black	225.0 (6.8)	326.7 (9.9)	30803576.6 (12.1)	5.3	2.2
Asian	69.0 (2.1)	186.8 (5.7)	15753631.6 (6.2)	4.1	0.5
Other	78.0 (2.4)	84.7 (2.6)	6424515.7 (2.5)	0.1	-0.1
Hispanic	145.0 (4.4)	508.1 (15.4)	43287920.6 (17.0)	12.6	1.6
Education					
less than high school	643.0 (19.5)	977.8 (29.6)	75414390.1 (29.6)	10.1	0
High school	86.0 (2.6)	301.9 (9.1)	24149374.4 (9.5)	6.9	0.4
some college	939.0 (28.4)	422.0 (12.8)	32035571.8 (12.6)	-15.8	-0.2
college	698.0 (21.1)	720.5 (21.8)	55744049.8 (21.9)	0.8	0.1
master	936.0 (28.3)	879.8 (26.6)	67392801.2 (26.5)	-1.8	-0.1
Working Status					
Working now	1484.0 (44.9)	1578.4 (47.8)	125668850.2 (49.3)	4.4	1.5
Self-employed	282.0 (8.5)	179.1 (5.4)	12777064.7 (5.0)	-3.5	-0.4
Temporarily laid off	10.0 (0.3)	8.3 (0.3)	634011.3 (0.2)	-0.1	-0.1
Retired	809.0 (24.5)	720.7 (21.8)	49420969.4 (19.4)	-5.1	-2.4
Student	74.0 (2.2)	1.3 (0.0)	89974.5 (0.0)	-2.2	0
Maternity leave	3.0 (0.1)	4.2 (0.1)	319553.8 (0.1)	0	0
Illness/Sick leave	10.0 (0.3)	14.5 (0.4)	1118538.0 (0.4)	0.1	0
Disabled	200.0 (6.1)	102.1 (3.1)	7693509.3 (3.0)	-3.1	-0.1
Other	220.0 (6.7)	475.4 (14.4)	38996757.2 (15.3)	8.6	0.9
Unemployed	210.0 (6.4)	217.9 (6.6)	18016959.0 (7.1)	0.7	0.5
State					
Alabama	44.0 (1.3)	49.8 (1.5)	3827913.6 (1.5)	0.2	0
Alaska	4.0 (0.1)	15.2 (0.5)	532716.4 (0.2)	0.1	-0.3
Arizona	94.0 (2.8)	61.1 (1.9)	5367806.8 (2.1)	-0.7	0.2

Arkansas	32.0 (1.0)	40.9 (1.2)	2320775.6 (0.9)	-0.1	-0.3
California	280.0 (8.5)	415.3 (12.6)	30580518.4 (12.0)	3.5	-0.6
Colorado	40.0 (1.2)	54.1 (1.6)	4580211.9 (1.8)	0.6	0.2
Connecticut	37.0 (1.1)	11.2 (0.3)	2852561.5 (1.1)	0	0.8
Delaware	14.0 (0.4)	6.0 (0.2)	792014.8 (0.3)	-0.1	0.1
District of Columbia	8.0 (0.2)	40.9 (1.2)	578767.7 (0.2)	0	-1
Florida	239.0 (7.2)	253.8 (7.7)	17524281.0 (6.9)	-0.3	-0.8
Georgia	116.0 (3.5)	38.5 (1.2)	8212982.6 (3.2)	-0.3	2
Hawaii	9.0 (0.3)	37.0 (1.1)	1057219.4 (0.4)	0.1	-0.7
Idaho	21.0 (0.6)	91.4 (2.8)	1401922.9 (0.6)	0	-2.2
Illinois	125.0 (3.8)	96.0 (2.9)	9754734.5 (3.8)	0	0.9
Indiana	46.0 (1.4)	37.0 (1.1)	5207777.0 (2.0)	0.6	0.9
Iowa	33.0 (1.0)	44.4 (1.3)	2444730.7 (1.0)	0	-0.3
Kansas	24.0 (0.7)	27.0 (0.8)	2207213.0 (0.9)	0.2	0.1
Kentucky	52.0 (1.6)	25.0 (0.8)	3465954.1 (1.4)	-0.2	0.6
Louisiana	38.0 (1.2)	90.7 (2.7)	3505606.8 (1.4)	0.2	-1.3
Maine	6.0 (0.2)	25.7 (0.8)	1112656.3 (0.4)	0.2	-0.4
Maryland	59.0 (1.8)	54.6 (1.7)	4700342.5 (1.8)	0	0.1
Massachusetts	71.0 (2.2)	84.9 (2.6)	5361482.8 (2.1)	-0.1	-0.5
Michigan	118.0 (3.6)	59.2 (1.8)	7867173.0 (3.1)	-0.5	1.3
Minnesota	48.0 (1.5)	52.1 (1.6)	4392346.5 (1.7)	0.2	0.1
Mississippi	18.0 (0.5)	22.6 (0.7)	2250410.5 (0.9)	0.4	0.2
Missouri	55.0 (1.7)	18.1 (0.5)	4769769.8 (1.9)	0.2	1.4
Montana	12.0 (0.4)	36.4 (1.1)	854044.4 (0.3)	-0.1	-0.8
Nebraska	16.0 (0.5)	21.1 (0.6)	1451867.1 (0.6)	0.1	0
Nevada	34.0 (1.0)	71.4 (2.2)	2461105.2 (1.0)	0	-1.2
New Hampshire	17.0 (0.5)	23.3 (0.7)	1117944.8 (0.4)	-0.1	-0.3
New Jersey	95.0 (2.9)	84.8 (2.6)	6964543.2 (2.7)	-0.2	0.1
New Mexico	11.0 (0.3)	56.2 (1.7)	1630415.7 (0.6)	0.3	-1.1
New York	405.0 (12.3)	141.0 (4.3)	15243413.0 (6.0)	-6.3	1.7
North Carolina	88.0 (2.7)	97.7 (3.0)	8319817.9 (3.3)	0.6	0.3
North Dakota	4.0 (0.1)	20.4 (0.6)	577208.2 (0.2)	0.1	-0.4
Ohio	128.0 (3.9)	95.4 (2.9)	9158296.4 (3.6)	-0.3	0.7
Oklahoma	36.0 (1.1)	42.6 (1.3)	1962390.2 (0.8)	-0.3	-0.5
Oregon	39.0 (1.2)	53.0 (1.6)	3418942.9 (1.3)	0.1	-0.3
Pennsylvania	144.0 (4.4)	69.6 (2.1)	10160813.9 (4.0)	-0.4	1.9
Rhode Island	15.0 (0.5)	31.1 (0.9)	851256.0 (0.3)	-0.2	-0.6
South Carolina	57.0 (1.7)	94.6 (2.9)	4107357.0 (1.6)	-0.1	-1.3
South Dakota	13.0 (0.4)	65.2 (2.0)	671772.7 (0.3)	-0.1	-1.7
Tennessee	67.0 (2.0)	104.9 (3.2)	5399537.5 (2.1)	0.1	-1.1

Texas	210.0 (6.4)	161.8 (4.9)	22047521.8 (8.7)	2.3	3.8
Utah	26.0 (0.8)	54.4 (1.6)	2044110.4 (0.8)	0	-0.8
Vermont	5.0 (0.2)	16.8 (0.5)	510680.9 (0.2)	0	-0.3
Virginia	88.0 (2.7)	50.0 (1.5)	6629083.2 (2.6)	-0.1	1.1
Washington	65.0 (2.0)	25.9 (0.8)	6023155.8 (2.4)	0.4	1.6
West Virginia	21.0 (0.6)	45.4 (1.4)	1418397.0 (0.6)	0	-0.8
Wisconsin	62.0 (1.9)	41.5 (1.3)	4594462.8 (1.8)	-0.1	0.5
Wyoming	13.0 (0.4)	45.4 (1.4)	448159.5 (0.2)	-0.2	-1.2
Metro county	2816.0 (85.3)	2758.6 (83.5)	222493432.8 (87.3)	2	3.8
Household Size					
1	698.0 (21.1)	609.6 (18.5)	47084861.9 (18.5)	-2.6	0
2	1155.0 (35.0)	1184.5 (35.9)	86861158.7 (34.1)	-0.9	-1.8
3	508.0 (15.4)	554.8 (16.8)	44465083.9 (17.5)	2.1	0.7
4	609.0 (18.4)	507.9 (15.4)	41341780.4 (16.2)	-2.2	0.8
5	246.0 (7.5)	263.8 (8.0)	21311897.3 (8.4)	0.9	0.4
more than 5	86.0 (2.6)	181.3 (5.5)	13671405.1 (5.4)	2.8	-0.1
Family Income					
Less than $10,000	205.0 (6.3)	307.5 (9.5)	9980755.3 (3.9)	-2.4	-5.6
$10,000 to $19,999	279.0 (8.6)	312.3 (9.7)	17761201.2 (7.0)	-1.6	-2.7
$20,000 to $29,999	298.0 (9.1)	334.6 (10.3)	21676442.2 (8.5)	-0.6	-1.8
$30,000 to $39,999	276.0 (8.5)	301.4 (9.3)	25273197.6 (9.9)	1.4	0.6
$40,000 to $49,999	243.0 (7.5)	288.6 (8.9)	19051200.4 (7.5)	0	-1.4
$50,000 to $59,999	250.0 (7.7)	292.5 (9.0)	20844810.6 (8.2)	0.5	-0.8
$60,000 to $99,999	786.0 (24.1)	770.7 (23.8)	60155849.0 (23.6)	-0.5	-0.2
$100,000 to $149,999	583.0 (17.9)	400.3 (12.4)	38001565.2 (14.9)	-3	2.5
$150,000 or more	339.0 (10.4)	226.8 (7.0)	41991165.9 (16.5)	6.1	9.5
Marital Status					
Married	1989.0 (60.8)	1794.1 (55.0)	130042853.3 (51.1)	-9.7	-3.9
Widowed	212.0 (6.5)	201.0 (6.2)	14914800.7 (5.9)	-0.6	-0.3
Divorced	407.0 (12.4)	383.8 (11.8)	25557773.8 (10.0)	-2.4	-1.8
Separated	54.0 (1.6)	62.3 (1.9)	4363383.0 (1.7)	0.1	-0.2
Never married	611.0 (18.7)	822.5 (25.2)	79857376.4 (31.3)	12.6	6.1

Note. Employing post-stratification weights significantly reduces the differences of survey estimates from the target estimates including marital status that was not considered in raking procedures.

Table S7. Summary of network characteristics before and after survey raking.

	unweighted	weighted	Diff. in percentage
N	41033	41034.8	
Network Size			
0	4499.0 (11.0)	5575.5 (13.6)	-2.6
1	16593.0 (40.4)	17002.7 (41.4)	-1
2	10507.0 (25.6)	9893.1 (24.1)	1.5
3	5267.0 (12.8)	4775.9 (11.6)	1.2
4	2308.0 (5.6)	2126.5 (5.2)	0.4
5	865.0 (2.1)	767.4 (1.9)	0.2
6	994.0 (2.4)	893.7 (2.2)	0.2
Relationship Composition (%)			
Parent	14	12.1	1.9
Spouse	29.5	32.6	-3.1
Child	8.8	9.6	-0.8
Sibling	8.9	8.7	0.2
Other family	6.1	6.4	-0.3
Coworker	0	0	0
Friend	23.8	21.6	2.2
Neighbor	1.4	1.4	0
Other	4.3	4.3	0
Communication channel (%)			
In-person	61.7	63.9	-2.2
Phone	44.4	43.3	1.1
Video	16.2	13.4	2.8
Text message	35.8	32.4	3.4
Email	8.3	7.4	0.9
SNS	10.4	8.5	1.9
Other channel	1.8	1.8	0
Homophily (i.e., % Same Alters)			
Sex	51.8	48.2	3.6
Race	85.5	83	2.5
Age	57.4	58.2	-0.8
Education	53.5	51.6	1.9
Partisanship	59.6	55.7	3.9
